# Device Identification Interoperability in Heterogeneous IoT Platforms †

**DOI:** 10.3390/s19061433

**Published:** 2019-03-23

**Authors:** Jahoon Koo, Se-Ra Oh, Young-Gab Kim

**Affiliations:** Department of Computer and Information Security, Sejong University, Seoul 05006, Korea; sigmao91@sju.ac.kr (J.K.); terious551@sju.ac.kr (S.-R.O.)

**Keywords:** Internet of Things, device identification, interoperability, IoT platform

## Abstract

With the continuous improvement of Internet of Things (IoT) technologies, various IoT platforms are under development. However, each IoT platform is developed based on its own device identification system. That is, it is challenging to identify each sensor device between heterogeneous IoT platforms owing to the resource request format (e.g., device identifier) varying between platforms. Moreover, despite the considerable research focusing on resource interoperability between heterogeneous IoT platforms, little attention is given to sensor device identification systems in diverse IoT platforms. In order to overcome this problem, the current work proposes an IoT device name system (DNS) architecture based on the comparative analysis of heterogeneous IoT platforms (i.e., oneM2M, GS1 ‘Oliot’, IBM ‘Watson IoT’, OCF ‘IoTivity’, FIWARE). The proposed IoT DNS analyzes and translates the identification system of the device and resource request format. In this process, resource requests between heterogeneous IoT platforms can be reconfigured appropriately for the resources and services requested by the user, and as a result, users can use heterogeneous IoT services. Furthermore, in order to illustrate the aim of the proposed architecture, the proposed IoT DNS is implemented and tested on a microcomputer. The experimental results show that a oneM2M-based device successfully performs a resource request to a Watson IoT and FIWARE sensor devices.

## 1. Introduction

Recently, the Internet of Things (IoT) has attracted significant social attention globally. Consequently, the research focusing on combining IoT technologies is ongoing in various fields such as smart home, smart car, smart grid, and healthcare. With the continuous improvement of IoT technologies, various IoT platforms are under development including oneM2M, OCF ‘IoTivity’, Apple ‘HomeKit’, Samsung ‘ARTIK’, Google ‘Brillo/Weave’, AllSeen Alliance ‘AllJoyn’, IBM ‘Watson IoT’, GS1 ‘Oliot’, and FIWARE. IoT Platforms are software that connect various devices, including a variety of sensors, access points, and data networks. Therefore, it is indispensable to provide interoperable services relevant to a diverse set of IoT platforms. However, most of the current IoT platforms share common limitations. Each IoT platform is developed with its own device identification (ID) system. That is, each IoT platform has a different request format for using services or resources provided by the device. Moreover, the device identifiers used in these request formats that also differ in format. Therefore, interworking between heterogeneous IoT platforms is challenging and deserve consideration. For example, in smart home environments, IoT devices are connected through a smart home hub. Consequently, these IoT devices must use the same ID system for interworking. Using only devices based on the same standard is a considerable limitation for the users as it cannot fulfill the goal of creating an IoT that connects every object. Moreover, despite the considerable research related to resource interoperability between heterogeneous IoT platforms, little attention has been given to sensor device ID systems in diverse IoT platforms. However, most of the IoT platforms use resource request statements that include the device identifier. In this study, we analyze resource and device ID systems of the representative IoT platforms (i.e., oneM2M; global standard 1 (GS1) ‘Oliot’; IBM ‘Watson IoT’; open connectivity foundation (OCF) ‘IoTivity’; and future internet ware (FIWARE)). Furthermore, we propose and implement an IoT device name system (DNS) architecture that converts resource requests into the respective format of each IoT platform. In order to implement this IoT DNS, we create a scenario in which a oneM2M device requests resources and services from a Watson IoT sensor device. The IoT DNS checks the request to identify the platform of the selected resource sent by the oneM2M client. Based on this result, the IoT DNS generates the request statement for the other platforms using the value stored when the device was registered. Our results show that the oneM2M client can transmit its request to the Watson IoT and FIWARE sensor device via Root DNS and control the latter’s LED sensor.

This paper is organized as follows. Section 2 analyzes the related work found in the literature dealing with the interworking between heterogeneous IoT platforms and provides a background on resource request formats of the four considered IoT platforms. Section 3 proposes the architecture and the algorithm of the IoT DNS. In Section 4, we implement a proof of concept of the IoT DNS and demonstrate its working principle on an example scenario. Section 5 concludes our work.

## 2. Background

This study selected five IoT platforms (i.e., oneM2M, GS1 ‘Oliot’, IBM ‘Watson IoT’, OCF ‘IoTivity’, and FIWARE) because these five platforms are well-known and make an important contribution to the IoT industrial environment. For example, these organizations provide relevant standards and solutions. Also, Watson IoT is being developed based on cloud computing and other selected platforms are being developed as open source projects.

oneM2M is an international organization working on machine-to-machine (M2M) standards and provides object communication requirements for IoT systems, architectures, application programming interface (API) specifications, and security solutions. GS1 is a private international organization aiming for standardization. It focuses on the standardization of product ID barcodes, electronic documents, electronic catalogs, etc., used in all industries including distribution and logistics. IBM Corporation is a global leader in information technology services and consulting, accounting for nearly 50% of the global computer market. The OCF is an organization that provides interoperability guidelines, standards development, and certification programs for the IoT industry. FIWARE is a smart city service platform developed and distributed by FI-PPP (public–private partnership) of European Union (EU) with 23 countries. FIWARE provides OpenStack-based cloud environments, open APIs, easy IoT connectivity, big data analysis, real-time media processing, and advanced features for user interaction. FIWARE also adopts the use of next generation services interface (NGSI) that unifies the representation of information. NGSI is a protocol developed by open mobile alliance (OMA) to manage context information. It is a simple RESTful API enabling to perform updates, queries, or subscribe to changes on context information.

### 2.1. Device Identification System of Various IoT Platforms

While the interworking between heterogeneous IoT platforms is an important issue, currently no solution is being clearly addressed. To solve this interworking problem, we analyzed the device ID systems used in each IoT platform [1] in our previous work. The device ID systems of the four considered IoT platforms are summarized in Table 1. It represents the feature and ID system format of selected IoT platforms.

**Figure 1:**
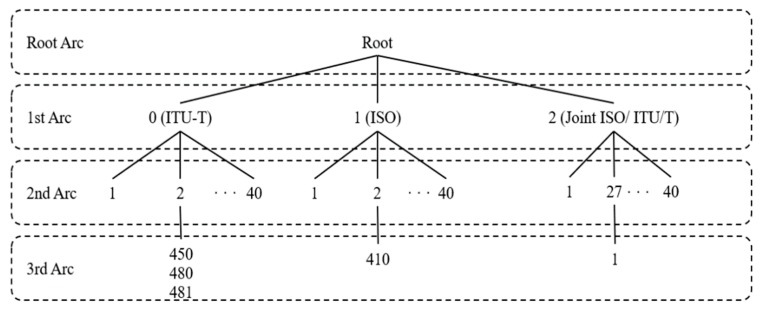
International OID tree.

**Figure 2:**
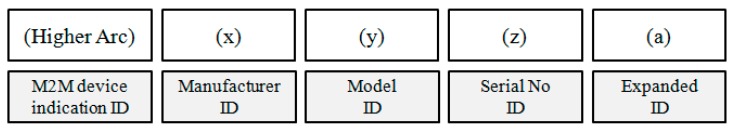
oneM2M standard object identifiers (OID).

The oneM2M uses object identifiers (OID) to identify devices. The OID are object identifiers organized into tree structures specified by the abstract syntax notation (ANS.1) and jointly developed by ITU-T and ISO/IEC, as shown in Figure 1. The international OID tree is referred to as the higher arc and is used as a prefix for the device ID system of oneM2M. As depicted in Figure 2, the higher arc is followed by ‘x’, ‘y’, ‘z’, where ‘x’ is the device manufacturer, ‘y’ is the device type, and ‘z’ is the device serial number [2].

The GS1 ‘Oliot’ also uses an OID-based ID key (namely the GS1) to identify devices and events. The OID assigned to GS1 is {2.51}. The first arc (2) contains the organization/institution code that represents the joint with ITU-T and ISO. The second arc (51) represents the GS1. {2.51} is followed by GS1 ID keys (1), GS1 supplementary data (2), GS1 business data (3), and GS1 technical data (4) as child nodes. GS1 ID keys (1) are used as device identifiers in the GS1, where the OID is {2.51.1}. The child nodes of {2.51.1} can have 10 key types, which are shown in Table 2. 

**Figure 3:**
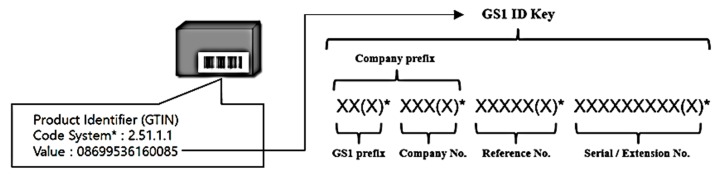
GS1 ID key value.

GS1 ID keys have various types depending on their usages, such as the global trade item number (GTIN) and serial shipping container code (SSCC). The GS1 ID key types can be identified by the field or application of the device. Individual devices can be identified through additional values. These GS1 ID key values include the company prefix, serial number, etc., as shown in Figure 3 [3,4,5,6].

The ID system of the IBM ‘Watson IoT’ identifies individual devices with unique client IDs. The client IDs are formatted by the client type. The client type is divided into application, expandable application, device, and gateway. The format of each client ID is shown in Table 3. The client ID used to identify each device has the following format: d:orgID:deviceType:deviceID. In this format, orgID is the user’s own organization ID. In order to register the device on the IBM Watson IoT platform, the user needs his own organization. IBM provides the orgID when the user registers his own account. Generally, IBM assigns a random six-digits-long string to every user. The deviceType is the type or model of the device. The deviceID is the serial number of the device [7].

The OCF ‘IoTivity’ identifies all resources, including the device, resource, etc., with the value of the resource type (‘rt’). Among them, the device is identified by using the ‘rt’ and an additional value referred to as the device identifier (‘di’). The ‘rt’ value can have an ‘oic.wk.d’ or ‘oic.d.[*]’. The ‘oic.d.[*]’ represents the specified device attribute. Currently, the ‘rt’ value is assigned the device type of the field for smart home applications and health care applications [8,9,10,11,12].

The device ID system of FIWARE is based on NGSI standard. FIWARE uses ‘entity id’ and ‘entity type’ to identify a specific entity. These entities also include each device connected to FIWARE, and the device ID and device type that identify each device can be set to ‘entity id’ and ‘entity type’. ‘Entity id’ and ‘entity type’ can be set freely by the user at the time of creation. There is no specific restriction on the format, but ‘entity id’ should be unique within the API and some elements (i.e., <, >, “, ‘, =, ;, (, )) are forbidden [13].

As shown in Table 4, we analyzed the four platforms and created a mapping table to compare them. The attribution in this table is based on the oneM2M OID structure because our study focuses on the oneM2M platform. All elements in the device ID of the GS1 Oliot are mapped to the oneM2M OID. However, the device IDs of the IBM Watson IoT, OCF IoTivity, and FIWARE do not use the device manufacturer and an expanded ID. Consequently, these values are mapped to n/a values. 

### 2.2. Resource Identification System of the Various IoT Platforms

Most IoT platforms use the device ID in the request statement. The subsection analyzes the resource request format of each heterogeneous IoT platform.

#### 2.2.1. oneM2M

**Figure 4:**
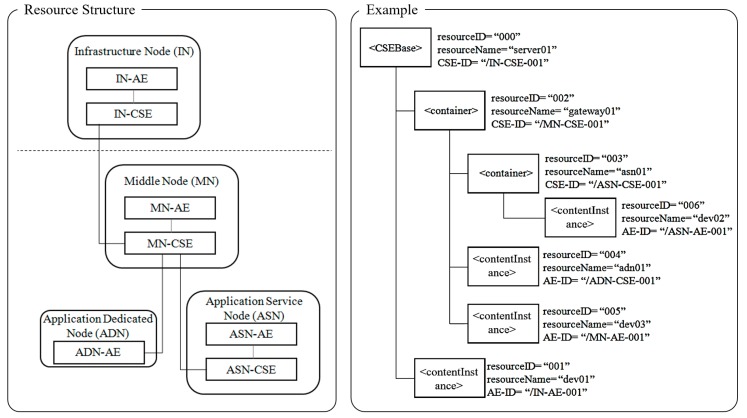
oneM2M resource structure.

The resource architecture of oneM2M is shown in Figure 4. This architecture consists of an infrastructure node (IN), middle node (MN), application service node (ASN), and an application dedicated node (ADN). Except for the ADN, every other node has a common service entity (CSE). The CSE has a resource structure with a tree format. In the oneM2M infrastructure, each resource has a resource ID and resource name. The latter two are used to request resources. For example, in order to request the ASN-AE with a specific resource ID (i.e., 006) and resource name (i.e., dev02), the path format is ‘server01/gateway01/asn01/dev02’. Currently, the oneM2M standard does not use the device ID when it requests resources and services [2]. 

#### 2.2.2. GS1 ‘Oliot’

GS1 Oliot uses the electronic product code information services (EPCIS) to store and manage devices and resources in an event format. In order to identify and request these devices and resources, the following ID formats are used: ‘urn:epc:id:sgtin:[GS1 ID key]’. This format contains the type and value of the GS1 ID key.

#### 2.2.3. IBM ‘Watson IoT’

IBM Watson IoT uses the request format as shown in Table 5. It supports the appropriate format according to the type of the protocol, e.g., hyper-text transfer protocol (HTTP) or message queuing telemetry transport (MQTT). In both formats, {typeID}, {deviceID}, and {logicalInterfaceId} are used as identifiers. The latter three have the expressions shown in Table 6 [7].

#### 2.2.4. OCF ‘IoTivity’

The resource request format used by the OCF IoTivity is ‘ocf://<authority>/<path>?<Query>‘. In this format, the ‘di’ value is used for the authority. The ‘di’ value uses the universally unique identifier (UUID) and has 36 characters. For example, if the request of the OCF IoTivity is ‘GET ocf://<di> /a/room/1?if=oic.if.ll’, the resource path is ‘/a/room/1’ and follows the query statement [11].

#### 2.2.5. FIWARE

**Figure 5:**
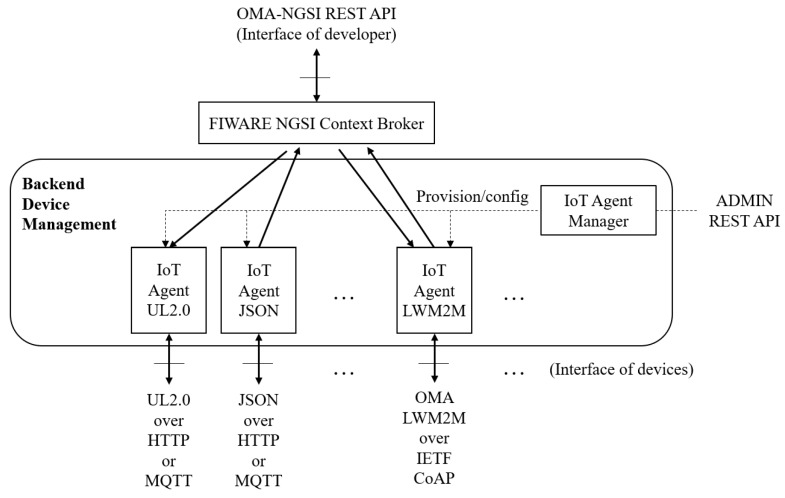
Interaction between Orion context broker and intelligence data advanced solution (IDAS) generic enablers (GEs).

The resource request format of FIWARE is different from each IoT agent. The IoT agent is a component that mediates between a set of devices using their own native protocols and an NGSI compliant context provider. In FIWARE, the intelligence data advanced solution (IDAS) generic enabler (GE) offers a wide range of IoT agents making it easier to interface with devices using the most widely used IoT protocols (e.g., lightweight M2M (LWM2M) over constrained application protocol (CoAP), javascript object notation (JSON), UltraLight over HTTP/MQTT, or OPC unified architecture (OPC-UA)) as shown in Figure 5. 

In this research, we use the NGSI-based HTTP resource request format and implement it in the testbed. The HTTP request format of FIWARE is {ip address}: 1026 / v2 / entities / {id}. {Ip address} is the IP of the device receiving the request, and FIWARE Orion uses port number 1026. {Id} is the identifier of the entity, and the attribution value is added to the format when users get or update the specific state information of the entity. The format for getting or updating specific status information is {ip address}: 1026 / v2 / entities / {id} / attrs / {attrsName} [13].

### 2.3. Related Work

oneM2M is an under-development interworking proxy entity (IPE) focusing on interworking with OCF. IPE converts the resources of OCF IoTivity into the oneM2M resource format and generates the resources in the gateway. The OCF IoTivity-based device connected to the oneM2M gateway gives the resource discovery results to the gateway at regular intervals. The gateway converts the results to the oneM2M resource tree structure and stores it in the gateway. A oneM2M device can request the OCF IoTivity resources that were converted to the oneM2M resource tree structure in the oneM2M gateway [14].

**Figure 6:**
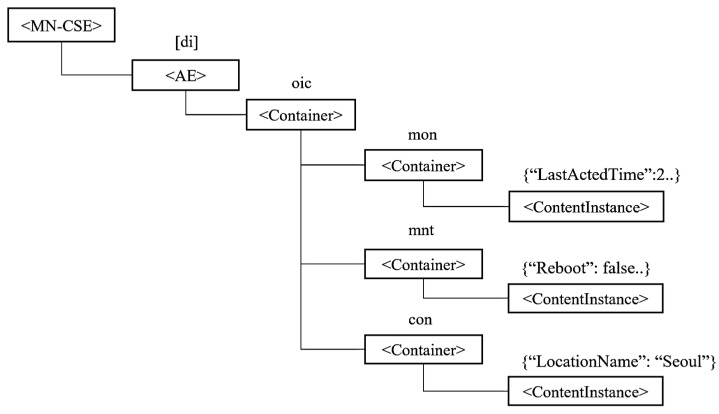
IoTivity resource mapping to oneM2M resource structure.

Wu et al. [15] presented an example for OCF IoTivity resource mapping to the oneM2M resource structure, as shown in Figure 6. In this example, ‘di’ is mapped to AE and ‘oic’ is mapped to the container under the MN-CSE tree. However, IPE converts resources in heterogeneous platforms to the oneM2M resource structures and, in addition, creates resources in the gateways. Hence, it is not efficient memory-wise at the gateways owing to the resources being newly created by only changing the structure. If large amounts of resources are created in the gateway, IPE requires a large amount of memory. Memory capacity is limited in the gateway and re-registering all resources is not efficient.

Kang et al. [16] proposed an ontology-based IoT framework that is based on the oneM2M standard to ensure the interoperability of heterogeneous IoT platforms. When a device is connected to the network, the IoT server registers the device as a oneM2M resource. For managing devices dynamically, the IoT server performs a health-check mechanism of the device. The data generated by the device registered is integrated into the oneM2M resource through monitoring. Moreover, the event about the data generated by the middleware is reported. However, the ontology-based IoT environment should be changed and rebuilt when the service requirements are changed or out of the set domain. It is also difficult to extract context information based on state information.

Tao et al. [17,18] built and managed a public cloud of private cloud-based platforms for each organization into a centralized cloud environment. These also aim to satisfy interoperability between heterogeneous platforms by applying ontology-based representation in public cloud. However, currently not all IoT platforms use the cloud and getting the information they need from a cloud-based platform is challenge. Further, additional research is required because there are many limitations in building cloud-based IoT environments.

Currently, interoperability between heterogeneous IoT platforms is under development by various research groups. However, most interoperability studies do not focus on device identifiers. As described previously, many related studies propose a solution by applying the IPE method specified by the oneM2M standard organization. The IPE, which is under development and is implemented on the oneM2M platform, creates and uses the resources of the heterogeneous platform by converting the format to the oneM2M resource format in the gateway. If large amounts of resources are created in the gateway, IPE requires a large amount of memory. Memory capacity is limited in the gateway and re-registering all resources is not efficient. In this study, we proposed a solution to these problems. We analyzed the resource request formats and device ID systems used in heterogeneous IoT platforms and selected several IoT platforms, such as the oneM2M, GS1 ‘Oliot’, IBM ‘Watson IoT’, OCF ‘IoTivity’, and FIWARE for analysis. Each IoT platform uses a different ID system, including both device identifier and resource request format. However, the IoT DNS proposed in this work does not re-register resources of the heterogeneous platforms in the oneM2M format. It stores and manages the request format aimed at the database in the gateway. Therefore, the gateway is less dependent on device capabilities and the memory usage of the IoT DNS is far less significant than that of the IPE.

## 3. IoT Device Name System Architecture

Currently, the five IoT platforms selected in this paper use different standards. Consequently, identifying devices and resources belonging to the various platforms is challenging. Therefore, this study proposes an IoT DNS architecture to solve these challenges. In Section 3, an algorithm and corresponding pseudo code of the proposed IoT DNS is presented. 

The IoT platform is divided into two types: One that provides cloud services, and one that builds its own servers. We suppose that the local DNS 1 is part of an Oliot platform, the local DNS 2 is part of a oneM2M platform, the local DNS 3 is part of an IoTivity platform, and the local DNS 4 is part of an FIWARE. Each Local DNS performs resource discovery in regular intervals and sends the resource discovery results to the root DNS. The root DNS integrates, stores, and manages the resource discovery results received from each local DNS in a single database. In addition, the integrated resource discovery results are transmitted to each local DNS at regular intervals. The integrated resource discovery results are provided to the client with only limited information. An example of a database managed by the root DNS and an example of the interface provided by local DNS to the client is as follows. The interface provided by the local DNS to the client includes three columns such as ‘Number’, ‘Resource’, and ‘IP’. ‘Number’ is the resource number in the database managed by the root DNS. It is used whenever a user selects that specific resource. ‘Resource’ is the resource or service name. ‘IP’ is the IP address or physical location of the device. It helps the user to identify the resource in more detail. The database managed by the root DNS includes seven columns: ‘Resource Number’, ‘Platform Number’, ‘Resource Name’, ‘Device ID’, ‘Resource Path’, ‘Use-Token-Auth’, and ‘IP’. The ‘Resource Number’, ‘Resource Name’, and ‘IP’ values are the same as the ones of the interface provided by the local DNS. The ‘Platform Number’ is the type of the IoT platform. In this study, we suppose that the platform number 1 refers to oneM2M, 2 refers to GS1, 3 refers to Watson IoT, 4 refers to IoTivity, and 5 refers to FIWARE. ‘Device ID’ is the identifier of the resource’s device. ‘Resource Path’ is the logical location of the resource. It is used whenever the IoT DNS generates a new request statement. ‘Use-Token-Auth’ is the token value used by the Watson IoT platform. If the IoT DNS generates a request statement for the Watson IoT platform, it uses the ‘Token-Auth’ value for authentication. Local DNS processes make requests for devices of the same platform. The root DNS generates the appropriate request statements for devices from heterogeneous platforms.

**Figure 7:**
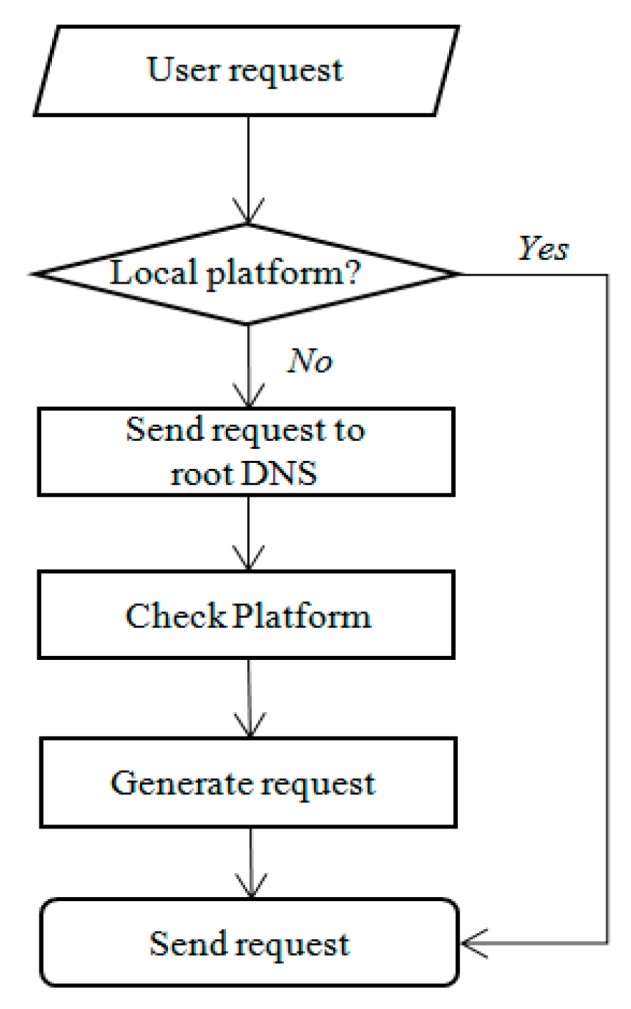
IoT device name system (DNS) algorithm.

**Figure 8:**
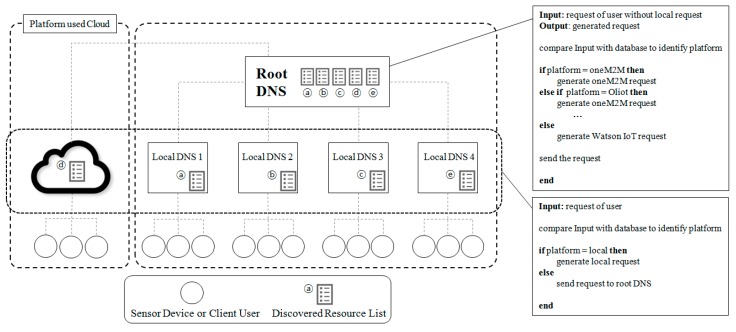
IoT DNS architecture.

Figure 7 shows the algorithms of the IoT DNS. A local DNS receives a user request from a client. If this request is compatible with the local system, the local DNS processes the request on the same platform. Otherwise, i.e., if the request is not compatible with the local system, the local DNS sends the request to the root DNS. The root DNS checks the platform and generates the request statements for the heterogeneous platform. A pseudo code of the root DNS and a local DNS for the above-described algorithm is shown in Figure 8.

## 4. Proof of Concept

In this section, in order to illustrate the aim of the proposed IoT DNS architecture, the proposed IoT DNS is implemented and tested on a microcomputer. Furthermore, we show two scenarios (i.e., in scenario 1, the oneM2M device requests a service to the Watson IoT and, in scenario 2, the oneM2M device requests a service to the FIWARE) which show how the proposed architecture works between diverse IoT platforms. A more detailed description will be explained in the following subsections. 

### 4.1. Implementation Environments

This subsection describes the implementation environment of the IoT DNS, which is specified in Table 7. The scenario presented in this study consists of the root DNS, a oneM2M server, a oneM2M client, a sensor device based on the Watson IoT platform, a FIWARE broker, and a sensor device based on the FIWARE platform.

The root DNS, the oneM2M server, and the oneM2M client use python. In addition, javascript is used for the oneM2M environment implementation. This javascript source is used to build the Mobius server named ‘yellow turtle’ and the oneM2M client named ‘&Cube’. Mobius was developed by Korea Electronics Technology Institute (KETI). It is a platform based on oneM2M.&Cube is developed by KETI and it is a oneM2M-based client software. The present work uses Mobius following the example documentation published by KETI. A python requests module is used, which sends the requests in python. It uses a MySQL connector module named PyMySQL, which connects MySQL and python. 

**Figure 9:**
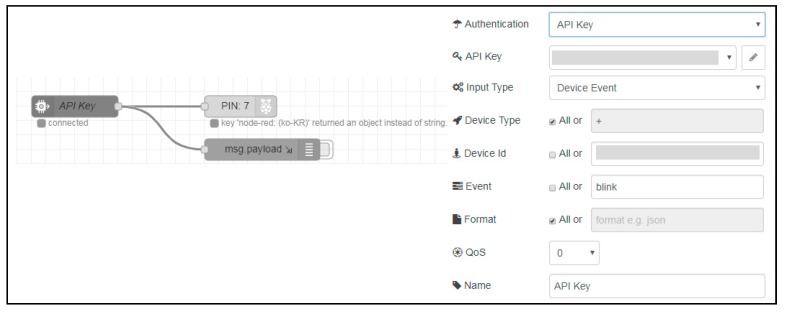
Node-RED configuration of the Watson sensor device.

In order to use the Watson IoT services, we registered a user account with IBM and were assigned a cloud. The Watson IoT-based sensor device receives requests through the IBM cloud. We have done the node configuration using Node-RED in a Watson IoT sensor device, as shown in Figure 9. Node-RED is a flow-based development tool for vision programming developed by IBM to wire hardware devices, APIs, and online services as parts of the IoT. As shown in Figure 9, the device receives the request with a satisfactory device ID and event named as blink. When setting up the node, the user is authenticated using the API key registered by the IBM cloud. 

**Figure 10:**
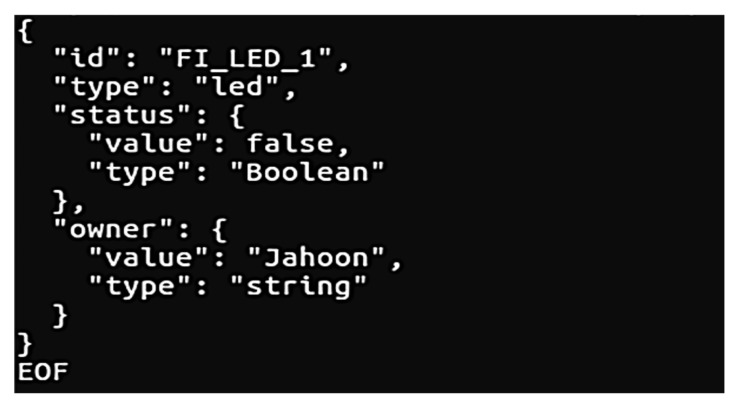
Implementation testbed.

In order to build the FIWARE environment, we use the Orion context broker that the user can register and manage the context elements through queries. As shown in Figure 10, we created the LED entity that ‘id’ status is FI_LED_1. This LED entity information is synchronized to FIWARE broker at regular intervals.

**Figure 11:**
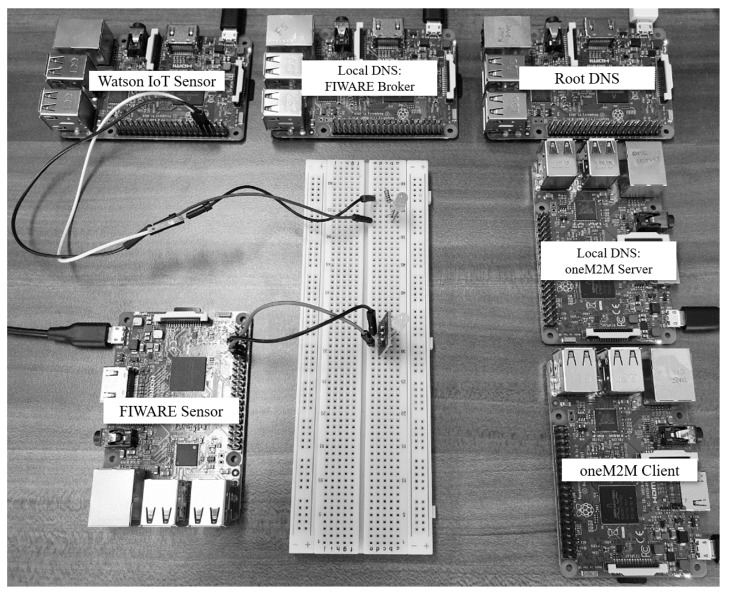
Implementation testbed.

Some elements in this implementation (i.e., the root DNS, oneM2M server, oneM2M client, Watson IoT sensor) are constructed using a raspberry Pi 3 Model b+, running the Raspbian stretch operating system, desktop version Nov. 2018. Other elements (i.e., FIWARE broker and FIWARE sensor) are running the Ubuntu MATE 16.04.2. The implementation testbed is shown in Figure 11.

### 4.2. Scenario

**Figure 12:**
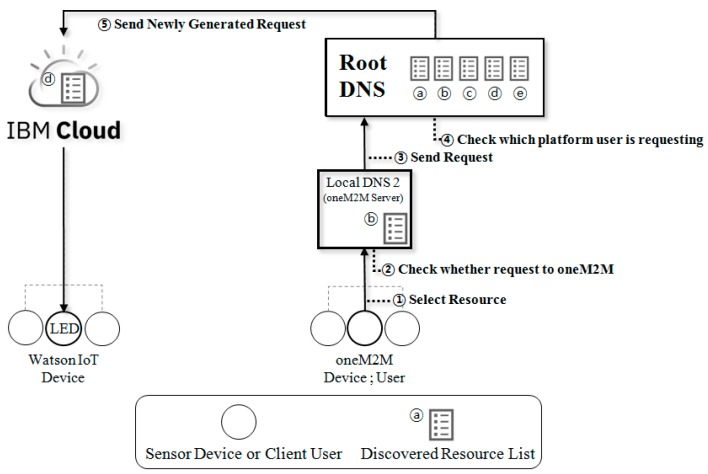
Scenario 1: A oneM2M request to a Watson IoT.

**Figure 13:**
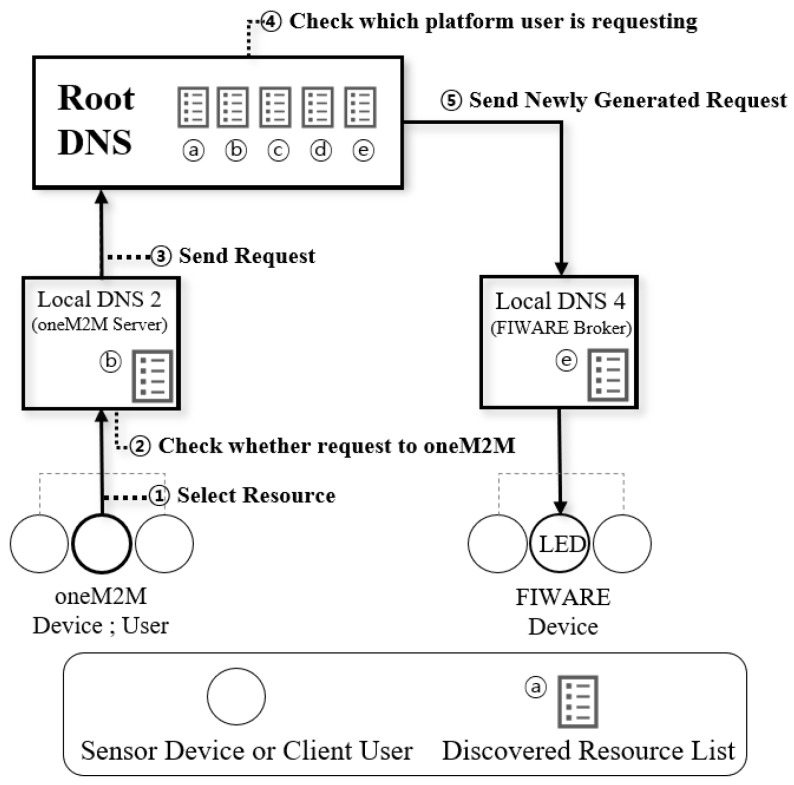
Scenario 2: A oneM2M request to a FIWARE.

This subsection describes the implemented scenario of the IoT DNS. The scenario supposes interworking between heterogeneous IoT platforms. As a case study, it is supposed that the user of the oneM2M client requests and controls the sensor (i.e., Watson IoT and FIWARE LED service). In advance, the oneM2M server (i.e., the local DNS) receives a list of all resources from the root DNS. When the oneM2M server and the client start communicating, the oneM2M server provides the user with a list of every resource. Scenario 1 (oneM2M device requests a service to Watson IoT sensor device) is shown in Figure 12 and scenario 2 (oneM2M device requests a service to FIWARE sensor device) is shown in Figure 13. The detailed description of the scenario is as follows:

1. The user of the oneM2M client selects the required resource named ‘LED:on’ from the resource list.
2. The oneM2M server checks the resource selected by the user in the database. The oneM2M server confirms that it is not a request to a oneM2M-based device and sends the request to the root DNS.
3. The root DNS checks the request received from the oneM2M server.
4. The root DNS identifies whether the request is for Watson IoT-based device or a FIWARE-based device.
5. The root DNS creates and sends a new request statement using the values in the database.

### 4.3. Implementation

**Figure 14:**
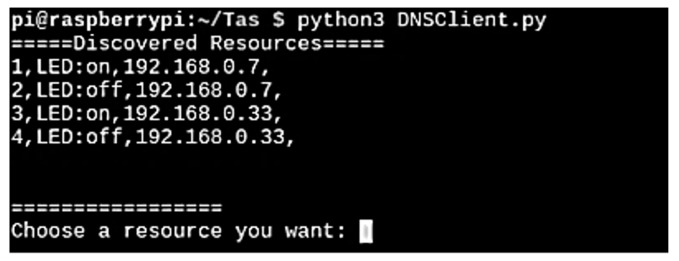
Example of the oneM2M client interface.

This subsection shows the IoT DNS testing based on the scenario described above. When the oneM2M client is connected to the oneM2M server, the user is provided with a list of resources available to the user. Figure 14 shows an example of a client interface. The example provides the resource number, resource name, and IP address. When the user selects the required service, the oneM2M client transmits the request to the connected oneM2M server.

**Figure 15:**
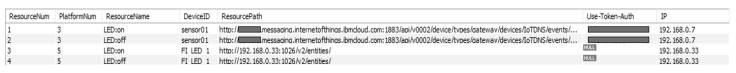
IoT DNS database example.

The oneM2M server compares the received resource number with the database and checks if it is a request to a oneM2M device. The first column in the database table, as shown in Figure 15, represents the resource number. The present study uses a configuration in which platform number 1 refers to the oneM2M platform, number 3 refers to the Watson IoT platform, and number 5 refers to the FIWARE platform. Since all services registered in the example table belong to number 3 and number 5 without number 1, the oneM2M server sends a request to the root DNS.

**Figure 16:**
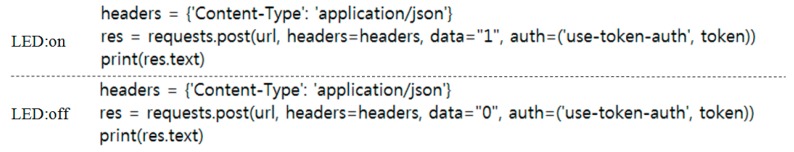
Requests code for LED on/off.

The root DNS identifies the request from the oneM2M server. In scenario 1, since it is a request to a Watson IoT device, the root DNS generates a request statement used by the Watson IoT platform. Figure 16 shows the code that generates the request statement using the Watson IoT resource request format.

**Figure 17:**
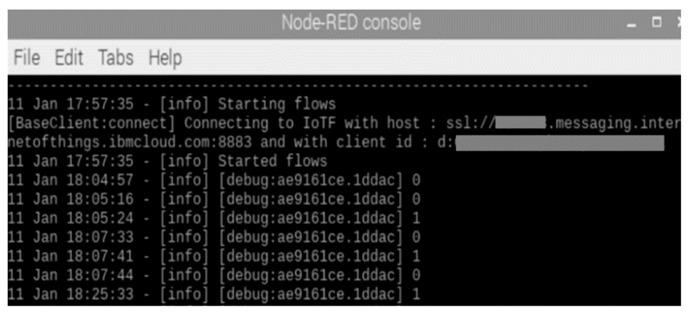
Success message of the LED request in Node-RED.

**Figure 18:**
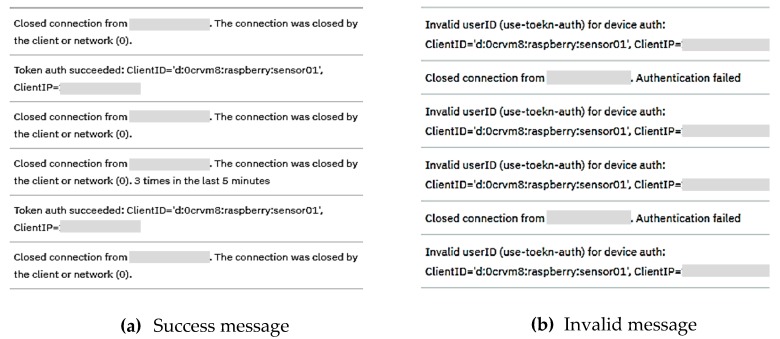
LED request message in IBM cloud.

If the root DNS successfully generates and sends the request to the Watson IoT, the user can control the LED sensor. As shown in Figure 17, if a request is successfully generated, it is presented through the Node-RED console window of the sensor device. As shown in Figure 18a, the user can check if the requests were successfully authenticated and received using the dashboard provided by the IBM cloud. If the root DNS uses an invalid value (e.g., device ID, path, token value), the user is presented with an ‘invalid value’ message, as shown in Figure 18b.

**Figure 19:**
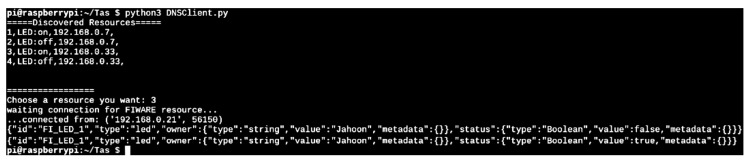
Status information of the FIWARE.

In scenario 2, if the root DNS identifies the request from the oneM2M server, since it is a request to a FIWARE device, the root DNS generates a request statement used by the FIWARE platform. If the root DNS successfully generates and sends the request to the FIWARE, the user can get and update the status of LED sensor. Then, if the user selects the LED:on, the root DNS gets the status of LED sensor and sends the update request that LED status is ‘on’ to FIWARE. Next, as shown in Figure 19, if a request is successfully generated, the result is presented through the console window of the sensor oneM2M client. The user also can check the current status and changed status.

## 5. Conclusion and Future Works

Currently, there is no clear solution for the interworking of devices belonging to heterogeneous IoT platforms. In this work, we proposed a solution to these problems, named IoT DNS, which converts requests from a oneM2M device to the appropriate request format for interworking between heterogeneous IoT platforms. We analyzed the resource request formats and device ID systems used in heterogeneous IoT platforms and selected several IoT platforms (i.e., oneM2M, Oliot, Watson IoT, IoTivity, and FIWARE). Furthermore, we created scenarios of implementation and carried out testing based on these scenarios. The scenarios included a user with a oneM2M device using a Watson IoT and FIWARE service. The testing results show that a oneM2M-based device successfully performed a resource requests to a Watson IoT and FIWARE sensor devices. 

Most related studies propose a solution based on the IPE method specified by the oneM2M standard organization. IPE converts resources in heterogeneous platforms to the oneM2M resource structures and, in addition, creates resources in the gateways. Hence, it is not efficient memory-wise at the gateways owing to the resources being newly created by only changing the structure. On the contrary, the IoT DNS stores only the logical location of the resources, not the entire resource, and directly requests the resource using this value when needed. Therefore, the memory usage of the IoT DNS is far less significant than that of the IPE.

In this work, we analyzed five platforms and presented a demonstration scenario involving the oneM2M, Watson IoT, and FIWARE platforms. However, in order to add a new IoT platform, we should manually register its ID system in IoT DNS. Moreover, cloud-based IoT platform (i.e., IBM Watson IoT) have limited functionality in the proposed IoT DNS because we are not able to directly modify it. Furthermore, security issues should be considered in the IoT DNS. For example, devices or users that want to use services in other platforms should be identified, authenticated, and authorized in advance. That is, security policies for secure access to resources in heterogeneous IoT platforms should be defined. Therefore, in our future work, we will expand the IoT DNS to automatically store the request format of worldwide IoT platforms and use the function provided by the cloud-based platform. In addition, our work will be extended by adding device authentication and authorization to the supported resource request processes. Consequently, these security policies will be properly applied to the IoT DNS to limit unauthorized access.

## Figures and Tables

**Table 1:** Comparison of four Internet of Things (IoT) platforms’ identification systems.

Platform	Feature	Identification Format
oneM2M	OID-based	OID (Higher Arc) ManufacturerID.DeviceTypeID.DeviceSerialNo
GS1 Oliot	OID-based	GS1 OID({2.51}).ID Keys(1).[ID Key Type], Value
IBM Watson IoT	Client ID	d:orgID:deviceType:deviceID
OCF IoTivity	Resource Type, Device ID	[di], rt:oic.wk.d,oic.d.[*]
FIWARE	Entity Type, Entity ID	No specific restriction except some characters (e.g., <, >, etc.)

**Table 2:** GS1 identification key type.

OID	ID Key Type	Name
2.51.1.1	GTIN	Global Trade Item Number
2.51.1.2	SSCC	Serial Shipping Container Code
2.51.1.3	GLN	Global Location Number
2.51.1.4	GRAI	Global Returnable Asset Identifier
2.51.1.5	GIAI	Global Individual Asset Identifier
2.51.1.6	GDTI	Global Document Type Identifier
2.51.1.7	GSRN	Global Service Relation Number
2.51.1.8	GSIN	Global Shipment Identification Number
2.51.1.9	GINC	Global Identification Number for Consignment
2.51.1.10	GCN	Global Coupon Number

**Table 3:** Type of Watson IoT client ID.

Client Type	ID Format
Application	a:orgID:appID
Expanded Application	A:orgID:appID
Device	d:orgID:deviceType:deviceID
Gateway	g:orgID:typeID:deviceID

**Table 4:** Mapping table for the device IDs of various IoT platforms.

Platform	X	Y	Z	a
oneM2M	Manufacturer ID	Model ID	Serial No ID	Expanded ID
GS1 Oliot	Company Prefix	Reference No	Serial No	Extension No
IBM Watson IoT	n/a	Device Type	Device ID	n/a
OCF IoTivity	n/a	rt: oic.d.[*]	di	n/a
FIWARE	n/a	Entity Type	Entity ID	n/a

**Table 5:** Watson IoT resource request format.

Protocol	Format
MQTT	iot-2/type/${typeId}/id/${deviceId}/intf/${logicalInterfaceId}/evt/state
HTTP	GET/device/types/{typeId}/devices/{deviceId}/state/{logicalInterfaceId}

**Table 6:** Request identifier parameter.

Parameter	Description
typeID	The device type identifier.
deviceID	The device identifier.
logicalInterfacedID or alias	The identifier created for the logical interface or the user-specified alias name.

**Table 7:** Specifications of the implementation environment.

		Root DNS	oneM2M Server (Local DNS)	oneM2M Client	Watson IoT Sensor	FIWARE Broker	FIWARE Sensor
Source Language		Python	Python Javascript	Python Javascript	n/a	Python C++	Python C++
Used Module & Tool		Python request module: Python requests version: 2.12.4 MySQL connector module: PyMySQL version: 0.9.3					
		Node-RED	Node.js Mobius	Node.js & Cube	Node-RED	Orion Context Broker	Orion-Client
Device	Name	Raspberry Pi 3 Model B+					
	CPU	1.4 GHz ARM Cortex-A53 MP4					
	OS	Raspbian Stretch with desktop version: Nov. 2018				Ubuntu MATE 16.04.2	
Additional Device		n/a	n/a	n/a	LED sensor	n/a	LED sensor
